# The cost-effectiveness of a new disease management model for frail elderly living in homes for the elderly, design of a cluster randomized controlled clinical trial

**DOI:** 10.1186/1472-6963-8-143

**Published:** 2008-07-07

**Authors:** Marijke Boorsma, Hein PJ van Hout, Dinnus H Frijters, Miel W Ribbe, Giel Nijpels

**Affiliations:** 1 Department of General Practice, EMGO-Institute, VU University medical center, Amsterdam, The Netherlands; 2 Department of Nursing Home medicine, EMGO-Institute, VU University medical center, Amsterdam, The Netherlands; 3 Westfriese Care Organization 'De Omring', Hoorn, The Netherlands

## Abstract

**Background:**

The objective of this article is to describe the design of a study to evaluate the clinical and economic effects of a Disease Management model on functional health, quality of care and quality of life of persons living in homes for the elderly.

**Methods:**

This study concerns a cluster randomized controlled clinical trial among five intervention homes and five usual care homes in the North-West of the Netherlands with a total of over 500 residents. All persons who are not terminally ill, are able to be interviewed and sign informed consent are included. For cognitively impaired persons family proxies will be approached to provide outcome information. The Disease Management Model consists of several elements: (1) Trained staff carries out a multidimensional assessment of the patients functional health and care needs with the interRAI Long Term Care Facilities instrument (LTCF). Computerization of the LTCF produces immediate identification of problem areas and thereby guides individualized care planning. (2) The assessment outcomes are discussed in a Multidisciplinary Meeting (MM) with the nurse, primary care physician, nursing home physician and Psychotherapist and if necessary other members of the care team. The MM presents individualized care plans to manage or treat modifiable disabilities and risk factors. (3) Consultation by an nursing home physician and psychotherapist is offered to the frailest residents at risk for nursing home admission (according to the interRAI LTCF). Outcome measures are Quality of Care indicators (LTCF based), Quality Adjusted Life Years (Euroqol), Functional health (SF12, COOP-WONCA), Disability (GARS), Patients care satisfaction (QUOTE), hospital and nursing home days and mortality, health care utilization and costs.

**Discussion:**

This design is unique because no earlier studies were performed to evaluate the effects and costs of this Disease Management Model for disabled persons in homes for the elderly on functional health and quality of care.

**Trail registration number:**

ISRCTN11076857

## Background

Publishing the design of a study and the results of the pilot is seen as useful by various publishers because of the possibility to compare the originally intended and hypothesized objectives and the final outcomes. Some authors mentioned that publishing the design of a study prevents publication bias of adverse or negative outcomes [1,2]. A positive effect of publishing a design article is prevention of such bias [1]. In addition, publishing the pilot results provides a better insight in the choices for particular instruments and interventions [1].

### Care problems of elderly in homes for the elderly

Persons in the homes for the elderly suffer greatly from (multiple) chronic diseases and associated disablement [3] Over the last decades, Dutch residents of homes for the elderly have become older and more disabled and show more and more resemblance with nursing home patients [4,5]. Primary care physicians (PCP) are responsible for the medical treatment of persons living in homes for the elderly. However, primary care physicians are often unable to handle the complex medical problems [6,7]. Many health problems go unnoticed by the primary care physicians [8].

### Disease Management Model

The Disease Management Model is based on 3 elements: coordination of care, guiding of the care process and empowerment of the patient [10]. This model is strongly recommended to improve the health and quality of life of the chronically ill [11-13]. Beneficial effects of disease management were reported among stroke patients and among diabetes mellitus type 2 patients [14-16]. However no studies were performed yet to evaluate the effects on functional health, quality of care and the cost-effectiveness of disease management for disabled persons in homes for the elderly. We use the concept of disease management but not focused on the diseases level but on the disabilities and handicaps they cause.

Already in 1995 the National health Council of the Netherlands stressed the importance of improving the quality of care for chronic patients by a shared disease management of the health professionals involved, with clearly defined medical responsibilities, and the development of shared management protocols [17].

### The Disease Management Model in residential homes

In residential homes, implementation of the three elements of the Disease Management Model demands specific adaptation and agreement across the responsible players. For example, who is best suited to do the guidance and coordination? Primary care physicians are responsible but do not regard themselves suited for systematic management and long-term monitoring for chronic diseases and disabilities associated with frail health [9]. In residential homes, nurse helpers have daily contact with the frail elderly and are well positioned to coordinate the care. Appropriate care coordination demands up to date and state of the art medical and social input. Guidance and medical input is needed by regular contact with the PCP. However, PCP often skip team meetings as they can be at impractical times, or have unclear agenda's which greatly limits its potential value. To increase the quality of the team meetings, PCP should be present, and nurse helpers could be trained care to bring forward relevant medical observations. Also, a consultant such as a geriatrician or other old age physician may be invited to provide state of the art advice. This approach demands empowerment of residential personnel by systematic observation and effective communication with medical professionals.

In addition, the issue of patient empowerment can be problematic. For example, about half of the residents suffer from dementia which greatly limits the potential for patient empowerment.

### Chronic disorders and homes for the elderly in the Netherlands

In our aging population the number of persons with a chronic disease is expected to increase from 1994–2015 with 25–60% [18]. In the Netherlands there are about 110.000 residents in homes for the elderly [4]. Professional care is needed for 71% of the residents such as assistance with activities of daily living or mobility, nursing care (medication, wound care etc) and domestic help. Twelve percent of persons of 75 years and older live in a home for the elderly and 4% in a nursing home [19]

The quality of care in these homes is frequently discussed in national and international newspapers. The care organizations responsible for the quality of care given in homes for the elderly often do not have the tools to measure and improve quality of care. Scientific studies of quality of care for the elderly are rare.

### Costs

Aging is costly for health care systems. About one third of the health care expenditures in industrialized countries involves persons of 70 years and older. Elderly are massive consumers of medication and occupy most of hospital beds (3). Studies of comparable interventions and associated costs in residential homes are absent. Nevertheless, we reanalyzed two meta-analyses of Stuck 2002 and Elkan 2000 on preventive effects of home visits to community dwelling elderly and selected only studies that focused on frail elderly 12 of 27 trials [20,21]. Six of these studies that reported on costs, found that preventive visits or outreaching geriatric management reduced care costs [22].

Objectives for this article were to describe the design of an evaluation study on the clinical and economical effects of a new disease management model for residents in homes for the elderly.

## Methods

### Design

**Figure 1:**
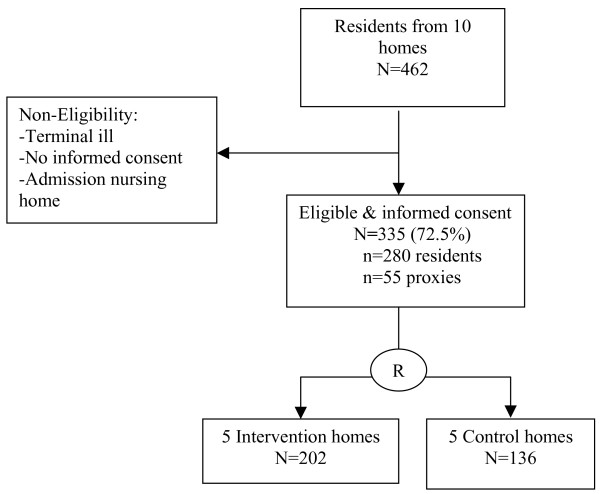
Flow chart of the design of PIKOV: Preventive effects of disease management on disabled persons within homes for the elderly, a cluster randomized controlled clinical trial.

A cluster randomized controlled clinical trial is carried out among five intervention homes and five usual care homes for the elderly in the north-west of the Netherlands that comprise over five hundred residents. There is a follow up of six months (Figure 1). The ethical committee of the VU medical center approved the study.

### Randomization

The randomization is carried out on home matched by percentage of psycho geriatric (i.e. cognitive impaired) residents. The care services and type of disability in homes with a high percentage of psycho geriatric patients are likely to differ depending on how many residents need psycho geriatric care. So, the homes were first ranked on percentage of psycho geriatric patients. The two homes with the highest percentage of psycho geriatric patients were than matched, and so on.

Next, we checked the risk of imbalance in numbers following Pocock's recommendations [23]. If the difference in number of intervention and control residents would be >15% (75 or more) the randomization should be repeated until the imbalance was 15% or less.

Homes were all ordered on the percentage psycho-geriatric patients and numbered from rank one to rank 10. In this way matched homes are ranked after each other, one having an even and the other an uneven number. Randomization was carried out using Pocock's first column in his random numbers table [23]. If the table's first number is even, the even number of first matched home is assigned the intervention. If the next table number is uneven, the uneven number of the next matched couple is assigned the intervention. And so on until all matched couples are assigned.

### Eligibility of residents

All residents were eligible except the terminally ill. Terminally ill is defined as death expected within six months. A family proxy of cognitive impaired persons was approached to provide outcome information.

### Procedure

All residents from the usual care homes as well as from the intervention homes receive an invitation letter and an informed consent form two weeks before the start of the study. If the resident is not able to understand the information and/or to sign the informed consent papers a close family member will be invited to participate and provide proxy information on the outcomes.

All eligible persons who sign an informed consent are going to be visited by an interviewer of the VU medical centre for an interview on their health and resource use. Table 1 provides an over-view of the measurements.

**Table 1:** Overview of outcomes and measurements in the study

Variable	Instrument	Baseline	6 months
*Primary outcome*			
a. Quality of Care	RAI-LTCF criteria	X	X
b. Quality Adjusted Life Years	Euroqol & thermometer	X	X
c. Functional health	COOP-WONCA & SF12	X	X
*Secondary outcome*			
d. Patient care satisfaction	Brief Quote on elderly Homes	X	X
e. Disability ADL-IADL	Groningen Activity Restriction Scale	X	X
f. Mood disorders	PRIME-MD	X	X
g. Hospital days	Checklist resource utilization Hospital records	X	X
h. Time to nursing home placement	Registration elderly home HIS		X
i. Time to mortality	Registration elderly home HIS		X
Economic outcomes			
Direct costs	Patient/family Interview Registration pharmacy Registration medical records		X
*Process outcomes*			
Adherence professionals to disease management protocol	Checklist		X
Adherence of patients to specific disease management recommendations	Checklist		X
Potential Effect Modifiers			
-Sociodemographics	Patient Interview	X	
-Health status (morbidity, medication)	Patient Interview Patient records	X	X
-House & personnel characteristics	Staff Interview	X	

### Intervention(s)

The Disease Management Model is based on 3 elements: coordination of care, guiding of the care process and empowerment of the patient [10]. A limitation of disease management for patients with multi-morbidity is the single-disease oriented perspective. Therefore in this project among elderly with mostly multiple morbidity, we choose an expanded multidimensional or biopsychosocial perspective which corresponds to the International Classification of Functioning, Disability and Health [24]. For our target population we defined disease management as improving or maintaining the functional health status by providing continuity of care, being patient oriented, generating multidimensional health data on residents, executed by appropriately trained professionals who design a shared disease management plan and is ICT supported.

In the intervention homes we will make disease management operational in the process of care in three sequential steps: 1. Firstly a three-monthly in-home systematic and computerized multidimensional assessment of all residents by staff (nurse) who systematically identifies the functional health status and care needs. For this purpose, the inter RAI LTCF instrument will be used [25].

The Resident Assessment Instrument (RAI) was originally designed as a minimum data set to assess the health needs of nursing home residents. For the homes for the elderly we use the inter RAI LTCF (Long Term Care Facility) version. The interRAI LTCF provides a comprehensive overview of the person's physical, psychological, behavioral and social status. Moreover it indicates a global level of care need which distinguishes persons who do not need care, from those who need personal care, home care, extramural home care or nursing home care. The computerized interRAI LTCF produces an easy and direct overview of problems in 18 areas that may need specific care planning. The identified problem areas guide the design of an optimal individualized care plan. In a multidisciplinary team, all disciplines involved in care for the resident, will participate in regular meetings in order to evaluate the interRAI LTCF findings and design and monitor the (tailor made) care-plan. The care plan aims to improve or maintain the functional health status and is focused at modifiable risk factors of the resident (Table 2).

**Table 2:** Case example RAI-LTCF assessment by nurse: triggered modifiable health risks

Problems and risks	Observed	Action now?
Delirium		
Cognition impairment /dementia		
Visual impairment	x	
Communication	x	
ADL-revalidation potential	x	x
IADL-more formal care needed		
Urinal incontinence	x	
Psychosocial wellbeing	x	x
Depression	x	
Behavior		
Social activities	x	
Falls	x	x
Nutrition	x	
Artificial nutrition		
Dehydration		
Dental health		
Skin problems and wounds	x	x
Psychotropic medication-walking problems		
Psychotropic medication- cognitive and behavioral problems		
Psycho medicaments and feeling unwell		
Fixation		

2. Secondly, the assessment outcomes are discussed in a multidisciplinary meeting (MM) in the homes with the primary care physician, nursing home physician, nurse, Psychotherapist and other involved disciplines. In the MM an individualized care plan is made to treat modifiable disabilities and identify and eliminate (when possible) risk factors.

3. Thirdly, a multidisciplinary consultation is offered to the frailest residents with complex health care problems. They are identified by the level of expected resource utilization [26].

In addition, the computerized interRAI LTCF also provides process-supporting information technology as well as indicators about the functioning and implementation of the care plans.

### Outcomes and measurements

Table 1 shows an overview of all outcome measures and instruments.

#### Primary outcomes are

1. Quality of care as measured with the risk adjusted criteria [27],

2. Quality Adjusted Life Years using health utilities is measured with the Euroqol [28,29],

3. Functional health is measured by COOP-WONCA charts [30,31] (Nelson 1983, Van Weel 1995) and Short Form 12 item version [32].

#### Secondary outcome measures are

4. Care satisfaction of residents is measured by the brief QUOTE, which wording was slightly adapted to fit the institutional setting [33].

5. ADL and IADL disability is measured by GARS [34].

6. (Days until) placement in a nursing home is surveyed and crosschecked at institutes.

7. (Acute) hospitalization is surveyed and cross-checked at the local hospital which covers 95% of all admissions in the region.

8. (Days until) mortality is checked in the administration of the homes.

#### Economic outcomes

9. Health care utilization data are collected by patient or proxy interview at baseline and patient records over 6 months.

### Sample size calculation

Sample size calculations are based on the expected effects of the intervention on the main outcome measures concerning quality of care and functional health. In the following sample size calculations we used an alpha of 0.05, power of 80% and inflation of 10% because of anticipated intra-cluster correlation in the homes for the elderly. Regarding health related quality of life, Cohen's D effect size ranged from 0.5 to 3.8 in our meta-analysis [22]. To detect a fair benefit, i.e. effect size = 0.5, a minimum of 64 persons is needed in each group [35]. For functional health and disability we anticipate on a comparable effect-size and consequently identical sample size. Furthermore if we assume a dropout rate of 15% during the 6 months follow-up we need to include at least 100/85 × 64 × 110% = 82 persons in each group.

### Data analysis

Effect analyses will be performed both on 'intention to treat' and per protocol principles. Differences between intervention and usual care patients at 6 months on the outcome measures (risk adjusted interRAI LTCF based Quality indicators, EuroQoL, functional health and disability) will be compared between the intervention and control group by both univariate and multivariate techniques. We will use the multivariate technique to adjust for possible differences in baseline scores and background variables between the intervention and control groups. Dropout and loss to follow up will be described. Potential effect-modification will be explored.

Especially, possible differential effects of disease management will be explored across residents with complex and simple health problems.

### Process evaluation

The process evaluation involves assessing the extent to which the intervention program is performed according to protocols, the nature of the recommendations made to the participants of the MM, compliance with these recommendations, physicians and therapists judgments about the intervention program and recommendations. Data on these topics are collected using structured registration forms during the intervention. Finally, semi-structured interviews will be held with the participating nurses, primary care physicians, and nursing home physicians at the end of the intervention period in order to record their experiences and opinions on the new disease management model.

### Economic evaluation

Cost data are collected by resident interview at base line, and at 6 months from a societal perspective and supplemented with resource use as registered within the home for the elderly. In case residents are cognitively impaired or not able anymore to be interviewed, proxies will be sought, preferably close family members. Only direct healthcare costs will be considered, because all patients have retired. Included cost categories are costs of consultations with the general practitioner, the nursing home physician and medical specialists, hospitalizations and admissions to the medical department of the nursing home and use of medication and medical aid. Medication data are retrieved from the centralized pharmacy files in the research region. Care consumption will be valued according to guidelines for economic evaluation in health care in the Netherlands [36,37].

#### Cost analysis

To compare costs between the two groups, confidence intervals for the difference in mean costs are calculated using bias-corrected and accelerated bootstrapping with 2000 replications [38].

#### Cost effectiveness analysis

For the cost-effectiveness analysis the difference in total costs between the intervention and usual care group are compared with the difference over 6 months in improvement of functional health and disability. In addition, a cost utility analysis will be done to assess the incremental costs per Quality Adjusted Life Years (QALY). QALY's are calculated by multiplying the utility based on EuroQol scores [29] with the amount of time a patient spent in this particular health state. Transitions between health states are linearly interpolated.

Uncertainty around the cost-effectiveness and cost-utility ratios is calculated using the bias-corrected percentile method (5000 replications) and presented in a cost-effectiveness plan [39]. The bootstrapped cost and effect pairs will also be used to calculate cost-effectiveness acceptability curves [40].

## Discussion

In this paper we described the design of a randomized cost-effective trial of the effect of Disease management on residents of homes for the elderly. This study holds several unique elements. The intervention concerns continuity of care and identification of care needs of the residents. The use of interRAI LTCF enables nurses to accurately diagnose the problems addressed within the complex clinical status of a frail elderly person. As a consequence, primary care physicians will be better informed about the health problems of their patients. This may enable effective disease management. Finally, to persons with complex problems a multidisciplinary consultation is offered by a nursing home physician.

The randomization on level of the homes for the elderly may be a weak point of the design as specific cultural habits of the homes will not be equally distributed over the two groups. On the other hand, randomization of homes will prevent contamination of the intervention to usual care homes.

The implementation of interRAI LTCF demands a great effort on the part of the organization and outcomes are dependent on good use of the instrument.

## Abbreviations

RAI- LTCF Resident Assessment Instrument – Long Term Care Facility; MM Multidisciplinary Meeting; SF12 Short Form 12 item version; QUOTE QUality Of care Through the patient's Eyes; GARS Groningen Activities Restriction Scale; ADL Activities if Daily Living; IADL Instrumental Activities if Daily Living

## Competing interests

The authors declare that they have no competing interests.

## Authors' contributions

MB and HvH drafted the paper. DF, MR and GN critically commented on the draft and all authors approved the final version.

## Pre-publication history

The pre-publication history for this paper can be accessed here:


